# In Memoriam

**Published:** 1888-07

**Authors:** 


### Editorial.

### *IN MEMORIAM.*

AUGUSTUS REGINALD DAVIDSON, M. D.,

Ex-President of the Buffalo Medical and Surgical Association.

Died May, 25, 1888, aged 43 years.

The ranks of this Society are again broken by the death of an honored member and beloved associate, who merited, by his unsullied character, acknowledged ability and professional attainments, the highest honor in his gift; and it is a beautiful illustration of the moral force of a pure life and noble heart that when fatal disease threatened, and vital power weakened, and life was imperiled, the heart of the profession and of the community throbbed in sympathy with the good physician, whose life was thus early sacrificed to the cause of human suffering.

It is fitting that this Society bear its testimony to his varied attainments, which were the fruits of patient labor and self-denial, to his achievements in medical science, and to the possibilities which were inseparable from his genius. We appreciate the character that was so perfect, and the life that was so pure and noble. We recognize the force and consistency of his religious example, which contributed so powerful an influence for good. We recall his friendship, which was so hearty and true, and we commend with gratitude the example of his life, which was guided by firm principle, by steadfast purpose, and by just and honorable motives.

In the prime of manhood, and in the very flush of his professional career, Dr. Davidson sacrificed his life to his zeal for the noble profession to which his superior abilities and scientific tastes peculiarly fitted him. He had lived long enough and accomplished sufficient to leave an indelible mark upon his profession and to secure a grateful remembrance in the hearts of a wide circle of devoted patients. His achievements in the special department of medical science to which he devoted his energies, have made a valuable and permanent addition to the treasures of medical science, and the world will profit by the recorded results of his indefatigable labors, which constitute a rich legacy of his love to the profession he honored by his associations and exalted by his service.

We rejoice that our brother was with us even for a little season. We mourn the sudden death, which forever removed him from us, with a sorrow which reaches our inmost hearts. But he was not taken unawares. His garments were always those of preparation. Men like him are ever ready to die. His life was beautiful, his influence was gracious, and his rest will be sweet. Having lived and worked worthily, and filled the measure of his day, he goes in triumph

> "To where, beyond these voices, there is peace."

